# Mid-Term Outcomes After Aortic Valve Replacement in Patients Under 70: A Comparative Study of INSPIRIS RESILIA Versus PERIMOUNT MAGNA EASE Bioprostheses

**DOI:** 10.1093/icvts/ivaf169

**Published:** 2025-07-25

**Authors:** Gabriel Saiydoun, Elie Nassar, Saadé Saadé, Chadi Aludaat, Sylvain Rubin, Ibrahim Alqdeimat, Vito Giovanni Ruggieri

**Affiliations:** Department of Cardiac Surgery, Pitié-Salpetrière University Hospital, Sorbonne University, 75013 Paris, France; Department of Cardiac Surgery, Henri Mondor University Hospital, 94000 Creteil, France; Department of Thoracic and Cardiovascular Surgery, Reims University Hospital, 51100 Reims, France; Université de Reims Champagne Ardenne, 51100 Reims, France; Department of Cardiac Surgery, Strasbourg University Hospital, 67000 Strasbourg, France; Department of Cardiac Surgery, Rouen University Hospital, 76031 Rouen, France; Department of Thoracic and Cardiovascular Surgery, Reims University Hospital, 51100 Reims, France; Université de Reims Champagne Ardenne, 51100 Reims, France; Department of Thoracic and Cardiovascular Surgery, Reims University Hospital, 51100 Reims, France; Department of Thoracic and Cardiovascular Surgery, Reims University Hospital, 51100 Reims, France; Université de Reims Champagne Ardenne, 51100 Reims, France

**Keywords:** aortic stenosis, aortic valve replacement, INSPIRIS RESILIA, PERIMOUNT MAGNA EASE

## Abstract

**Objectives:**

INSPIRIS RESILIA, launched in 2017, is a bioprosthetic aortic valve developed to improve durability and facilitate future valve-in-valve procedures. Despite its advanced design, many surgeons continue to use the PERIMOUNT MAGNA EASE valve, which has long-standing clinical validation. This study aimed to compare mid-term clinical and echocardiographic outcomes in patients under 70 undergoing aortic valve replacement with either prosthesis.

**Methods:**

We conducted a retrospective study of patients who underwent surgical aortic valve replacement between January 2018 and May 2023 at the University Hospital of Reims. The primary outcome was all-cause mortality at 1 year following surgical aortic valve replacement. Secondary outcomes included haemodynamic parameters, left ventricular ejection fraction, and major postoperative complications such as reintervention, stroke, pacemaker implantation, mediastinitis, transfusion, and new-onset atrial fibrillation.

**Results:**

A total of 300 patients were included: 52 received the INSPIRIS RESILIA valve and 248 received the PERIMOUNT MAGNA EASE valve. After matching, 52 patients from each group were compared. All-cause mortality at 3 years was 0% in the INSPIRIS group and 1.9% in the PERIMOUNT group. Mean transvalvular gradients were similar at 1 year (11.3 vs 11.2 mmHg) and 3 years (12.9 mmHg for both). Two cases of endocarditis-related reoperation occurred in the INSPIRIS group. No structural valve degeneration requiring surgery was observed. Postoperative aortic regurgitation was trivial or absent. Transfusion rates were lower in the INSPIRIS group (46.1% vs 69.2%, *P* = .017).

**Conclusions:**

INSPIRIS RESILIA and MAGNA EASE valves offer similar mid-term safety and performance in patients under 70 years of age.

## Graphical Abstract

**Figure:**
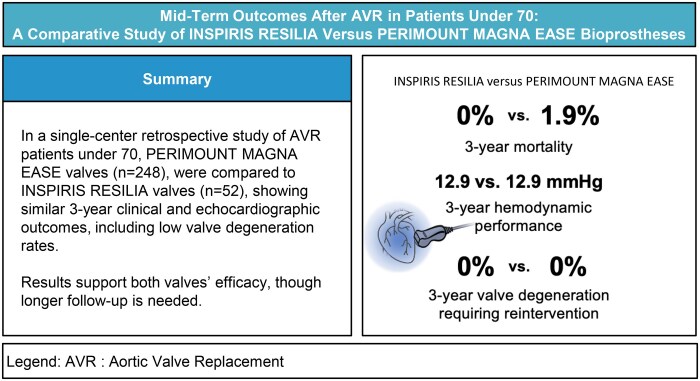

## INTRODUCTION

The selection of a bioprosthetic valve for surgical aortic valve replacement (AVR) is increasingly favoured, particularly in younger patients, due to the elimination of long-term anticoagulation and the possibility of future valve-in-valve (ViV) procedures. However, bioprosthetic valves remain subject to structural valve degeneration (SVD) over time, which may lead to reintervention.^1^ Consequently, choosing a durable valve with excellent haemodynamic properties is essential to improving mid- and long-term outcomes.

Introduced in 2017, the INSPIRIS RESILIA valve (Edwards Lifesciences, Irvine, CA, USA) (IR) is a tricuspid bovine pericardial bioprosthesis mounted on an expandable stent. It incorporates RESILIA tissue technology, designed to reduce calcification and improve tissue preservation through novel fixation and storage techniques.^2^

Early clinical data from both European and North American cohorts have demonstrated encouraging safety and haemodynamic results at 5 years.^3^^,^^4^ In particular, the COMMENCE trial showed low rates of SVD and reintervention, while maintaining stable gradients and minimal regurgitation up to 5 years.^3^ A recent systematic review further confirmed these mid-term findings, showing excellent valve durability up to 5.3 years of follow-up across multiple studies.^5^

Despite these promising results, most existing data are derived from observational or manufacturer-sponsored studies. Comparative, real-world evaluations remain essential to determine whether INSPIRIS RESILIA offers measurable advantages over standard bioprostheses, such as the widely used PERIMOUNT MAGNA EASE (Edwards Lifesciences, Irvine, CA, USA) (ME), known for its long-term durability and benchmark performance in surgical AVR.^1^^,^^4^

This study therefore aimed to compare the mid-term clinical and echocardiographic outcomes of patients under 70 years of age receiving either the INSPIRIS RESILIA or the PERIMOUNT MAGNA EASE bioprosthesis in a single-centre, real-world setting.

## METHODS

### Study population

All consecutive adult patients under 70 years of age who underwent primary surgical AVR with a bioprosthesis (IR or ME) at the University Hospital of Reims between January 2018 and May 2023 were included. Patients who underwent emergency or redo surgery, or received another type of prosthesis, were excluded. Both aortic stenosis and aortic regurgitation were considered valid indications for surgery, in accordance with the 2021 ESC/EACTS Guidelines for the Management of Valvular Heart Disease.^6^ The decision to implant a bioprosthesis in younger patients was based on multidisciplinary heart team evaluation, taking into account patient preferences, lifestyle, contraindications to anticoagulation, and the potential for future ViV procedures, as recommended by current European guidelines.

### Study approval

This single-centre study was conducted retrospectively in 2024, using pre-existing data from the EPICARD database. No prospective data collection was initiated at the time of surgery. This study was approved by the Institutional Review Board of the French Society of Thoracic and Cardiovascular Surgery in 2024 (IRB00012919-CERC-SFCTCV-2024-10-22_36523). All patients provided written informed consent for the use of their anonymized medical and echocardiographic data for research purposes, before inclusion in the EPICARD database. No biological material or data were collected or stored for future or indefinite use in biobanks; therefore, the WMA Declaration of Taipei is not applicable in this study.

### Procedure and postoperative patient follow-up

All surgeries were performed using the full sternotomy approach. A standardized surgical technique was used for all patients. Following excision of the native aortic valve, pledgeted U-shaped sutures were placed from the ventricular side to the aortic side at the level of the annulus. Valve sizing was performed using dedicated sizers, ensuring the largest size compatible with the annulus and patient’s body surface area was selected. The appropriate size was confirmed when the sizer passed without resistance and without obstructing the coronary ostia. The valve was then implanted and secured with standard manual knotting.

The selection of the ME and IR bioprostheses was based on their widespread clinical use and established safety profile in surgical AVR. The ME valve has been the standard prosthesis historically implanted in our institution, given its well-documented long-term durability and haemodynamic performance. In contrast, the IR valve was gradually introduced after its CE approval, based on promising theoretical advantages—particularly the use of RESILIA tissue technology designed to limit calcification and potentially enhance durability.

The choice between these 2 prostheses was not determined by patient-specific variables such as age, comorbidities, or annular size, but rather by surgical team preferences and institutional practice patterns. Each patient was included only once in the analysis, and there was no overlap between groups; patients received either an INSPIRIS RESILIA or a PERIMOUNT MAGNA EASE valve, but not both. **Figure S1** describes the study’s design.

### Demographic analysis and data collection

Basic population characteristics, along with clinical and echocardiographic data, were collected retrospectively after obtaining patient consent.

Surgical risk was assessed using the EuroSCORE II. Intraoperative data, including the type of native valve, choice of bioprostheses, bioprostheses size, and any associated procedures, were extracted from the operative report.

Echocardiographic follow-up data were collected at 4 specific times: preoperatively, at discharge, 1 year postoperatively, and 3 years postoperatively. The VARC3 definition is used for SVD.

### Outcome criteria

The primary outcome was all-cause mortality 1 year after AVR. Secondary outcomes included echocardiographic parameters such as the mean gradient before discharge, at 1 year, and at 3 years, as well as postoperative left ventricular ejection fraction. Clinical outcomes assessed were the need for reintervention, the incidence any major postoperative adverse event, including mediastinitis, postoperative stroke, postoperative transfusion, pacemaker implantation, and new-onset atrial fibrillation.

### Statistical analysis

Descriptive statistics were used to analyse data. Categorical variables were presented as absolute values and frequencies (%), and continuous variables as means with standard deviations or medians with interquartile ranges. Analyses were based on available cases. Group comparisons used t-tests or Mann-Whitney U-tests for continuous variables, depending on distribution, and Fisher’s exact tests for categorical variables. Kolmogorov-Smirnov tests assessed normality. Median follow-up was estimated using the reverse Kaplan-Meier method, which considers censoring and provides a robust estimate of potential follow-up time. Propensity score matching accounted for baseline differences. Scores were calculated by logistic regression adjusted for key variables, with a calliper of 0.1 for 1:1 matching. Overlap of propensity scores was graphically assessed to verify common support. Statistical approaches followed published guidelines on propensity score analysis and matched cohort comparison. Standardized mean differences (SMDs) were used to assess covariate balance after matching, with values <0.1 indicating good balance. Group comparisons in the matched cohort were performed using paired t-tests, Wilcoxon signed-rank tests, or McNemar’s tests. Survival analyses were performed using Kaplan-Meier estimates, and differences between groups were assessed with the log-rank test. Analyses were performed using R (v4.4.0; R Core Team 2024). A value of *P* < .05 was considered significant.

## RESULTS

### Patient characteristics

During the study period (January 1, 2018, to May 31, 2023), a total of **822 surgical** AVRs were performed in adult patients under 70 years. Among these, **757 patients received a bioprosthesis** and **65 received a mechanical valve**. Of the 757 bioprostheses implanted during the study period, all were either INSPIRIS RESILIA or PERIMOUNT MAGNA EASE valves; among them, 300 were implanted in non-emergency patients under 70 years of age, including 52 INSPIRIS RESILIA and 248 PERIMOUNT MAGNA EASE valves. The median follow-up was 29 months [15-47], and there was no difference in the follow-up between the 2 groups (ME: 27 months [17-43] vs IR: 29.5 [14-47]; *P* = .49).

The clinical characteristics of the 2 groups, both unadjusted and adjusted for propensity scores, are presented in **Table 1**. All SMDs were <0.1 in the adjusted group as illustrated in the love plot (**Figure S2**).

**Table 1.** Preoperative Clinical and Echocardiographic Characteristics in the Unadjusted and in the Propensity-Matched Groups

Variable	Unadjusted			Propensity-matched		
	INSPIRIS RESILIA (n = 52)	PERIMOUNT MAGNA EASE (n = 248)	*P-*value	INSPIRIS RESILIA (n = 52)	PERIMOUNT MAGNA EASE (n = 52)	*P-*value
**Male**	37 (71.2)	194 (78.2)	.3	37 (71.2)	44 (84.6)	.156
**Age**	61.6 (5.3)	64.6 (5.1)	**<.001**	61.6 (5.3)	66.4 (4.0)	**<.001**
**BMI**	29.3 ± 5.1	28.7 ± 5.3	.4	29.3 ± 5.1	29.0 ± 4.9	.769
**Hypertension**	34 (65.3)	183 (73.7)	.2	34 (65.3)	42 (80.8)	.122
**Dyslipidaemia**	29 (55.8)	145 (58.4)	.7	29 (55.8)	35 (67.3)	.314
**EuroSCORE II**	1.55 ± 2.10	1.38 ± 1.23	.069	1.55 ± 2.1	1.59 ± 0.991	.914
**COPD**	10 (19.2)	55 (22.1)	.6	10 (19.2)	14 (26.9)	.485
**Tobacco use**	10 (19.2)	62 (25)	.6	10 (19.2)	18 (34.6)	.19
**Dialysis**	0 (0)	1 (0.4)	.9	0 (0)	0 (0)	–
**Peripheral vascular disease**	5 (9.6)	66 (26.6)	**.009**	5 (9.6)	19 (36.5)	**.002**
**Type 2 diabetes mellitus**	17 (32.7)	70 (28.2)	.5	17 (32.7)	17 (32.7)	.99
**Prior atrial fibrillation**	3 (5.8)	35(14.1)	.4	3 (5.8)	17 (32.7)	**.001**
**Prior stroke**	0 (0.0)	10 (4)	.29	0 (0.0)	2 (3.8)	.475
**Pacemaker implantation**	1 (1.9)	6 (2.4)	.9	1 (1.9)	0 (0.0)	1.000
**Prior myocardial infarction**	0 (0)	5 2.0)	.6	0 (0.0)	3 (5.8)	.241
**Prior coronary angioplasty**	2 (3.8)	39 (16)	**.023**	2 (3.8)	17 (32.7)	**<.001**
**NYHA class III/IV**	11 (21.1)	54 (21.8)	.9	11 (21.1)	16 (30.7)	.25
**Endocarditis**	4 (7.7)	9 (3.6)	.3	4 (7.7)	5 (9.6)	.99
**Isolated AVR**	42 (80.8)	179 (72.1)	.2	42 (80.8)	35 (67.3)	.180
**Redo**	3 ( (5.8)	4 (1.6)	.1	3 (5.8)	0 (0.0)	.241
**BAV**	27 (51.9)	122 (49.1)	.7	27 (51.9)	25 (48.1)	.845
**Aortic insufficiency (grade II/III/IV)**	13 (29.4)	48 (19.35)	.7	13 (29.4)	8 (15.3)	.3

Abbreviations: AVR, aortic valve replacement; BAV, bicuspid aortic valve; BMI, body mass index; COPD, chronic obstructive pulmonary disease; IVS, interventricular septum; NYHA, New York Heart Association.**Bold values** indicate statistically significant differences (P < 0.05).

Approximately 77% of patients were male. Patients in the IR group were younger, with a mean age of 61.6 (5.3) versus 66.4 (4.0) years in the ME group (*P *< .001). The clamping time was comparable between the 2 groups, while the total cardiopulmonary bypass time was significantly higher in the ME (109.51 ± 35.05) than in the IR group (93 ± 22) (*P *= .004).

There was a higher rate of isolated AVR in the IR (*n* = 42, 80.8%) versus ME groups (*n* = 35, 67.3%) (*P *= .180), and 3 redo surgeries in the IR group compared to none in the ME group after adjustment, although these 2 parameters were not statistically significant.

Preoperative echocardiographic and intraoperative parameters are presented in **Tables 1** and **2**. It should be noted that the 2 groups were comparable across the different measurements, with the percentage of bicuspid aortic valves being reapproximate 50% of the study population.

**Table 2.** Intraoperative Data and Associated Procedures in the Unadjusted and in the Propensity-Matched Groups

Variable	Unadjusted			Propensity-matched		
	INSPIRIS RESILIA (n = 52)	PERIMOUNT MAGNA EASE (n = 248)	*P-*value	INSPIRIS RESILIA (n = 52)	PERIMOUNT MAGNA EASE (n = 52)	*P-*value
**Prosthesis size**			**<.001**			**.006**
**19**	1 (1.9)	5 (2.0)		1 (1.9)	1 (1.9)	
**21**	17 (32.7)	30 (12.1)		17 (32.7)	4 (7.7)	
**23**	26 (50.0)	100 (40.03)		26 (50)	28 (53.8)	
**25**	5 (9.6)	90 (36.3)		5 (9.6)	14 (26.9)	
**27**	3 (5.8)	16 (6.5)		3 (5.8)	4 (7.7)	
**29**	0 (0)	7 (2.8)		0 (0)	1 (1.9)	
**Type of associated procedure**						
**Coronary artery bypass grafting**	9 (17.3%)	62 (25%)	.25	9 (17.3%)	8 (15.4%)	.79
**Ascending aorta replacement**	4 (7.7%)	20 (8.1%)	.88	4 (7.7%)	4 (7.7%)	>.99
**Mitral or tricuspid valve repair**	2 (3.8%)	9 (3.6%)	.92	2 (3.8%)	2 (3.8%)	>.99
**Left atrial appendage closure**	2 (3.8%)	8 (3.2%)	.73	2 (3.8%)	2 (3.8%)	>.99
**Other procedures**	1 (1.9%)	6 (2.4%)	.66	1 (1.9%)	1 (1.9%)	>.99

**Bold values** indicate statistically significant differences (P < 0.05).

### Early postoperative results

Data concerning immediate postoperative follow-up and echocardiography results before hospital discharge are presented in **Table 3**.

**Table 3.** Postoperative Outcomes in the Unadjusted and in the Propensity-Matched Groups

	*Unadjusted*			*Propensity-matched*		
	INSPIRIS RESILIA (n = 52)	PERIMOUNT MAGNA EASE (n = 248)	*P-*value	INSPIRIS RESILIA (n = 52)	PERIMOUNT MAGNA EASE (n = 52)	*P-*value
Early reintervention	5 (9.6)	19 (7.7)	.6	5 (9.6)	8 (15.3)	.4
Postoperative bleeding	1 (1.9)	11 (4.4)	.7	1 (1.9)	3 (5.8)	.6
Postoperative PM	2 (3.8)	7 (2.8)	.7	2 (3.8)	2 (3.8)	>.9
Postoperative atrial fibrillation	7 (13)	38 (15)	.7	7 (13)	6 (12)	.8
Mediastinitis	2 (3.8)	3 (1.2)	.2	2 (3.8)	1 (1.9)	>.9
Postoperative stroke	0 (0)	1 (0.4)	>.9	0	0	–
RedoAVR	2 (3.8)	4 (1.6)	.3	2 (3.8)	0 (0)	.5
Transfusion	24 (46.1)	139 (56)	.2	24 (46.1)	36 (69.2)	**.017**
Length of stay (days)	7 [6-10]	7 [6-9.75]	>.9	7 [6-10]	8 [6-10]	.29
**Echocardiographic outcomes**						
Postoperative LVEF (%)	60 ± 6	58 ± 8	.092	60 ± 6	58 ± 8	.071
Postoperative mean gradient (mmHg)	11.0 ± 3.4	10.9 ± 3.6	.8	11.0 ± 3.4	10.58 ± 2.85	.6
Postoperative AI (grade I)	0 (0%)	5 (2.1%)	.6	0 (0%)	1 (2.0%)	>.9

Abbreviations: AI, Aortic insufficiency; AVR, aortic valve replacement; LVEF, left ventricular ejection fraction; PM, PaceMaker.

**Bold values** indicate statistically significant differences (P < 0.05).

The postoperative transfusion rate of any blood product was significantly higher in the ME group (69.2%) then in the IR group (46.1%) (*P *= .017). The early reoperation rates were 15.3% in the ME group and 9.6% in the IR group (*P* = .4).

The occurrence of postoperative atrial fibrillation and mediastinitis did not differ significantly between the groups. The average length of stay was 11 ± 9 days in the IR group and 8 ± 5 days in the ME group (*P *= .10).

The left ventricular ejection fraction (IR, 60% ± 6; ME, 58% ± 8; *P *= .071) and mean postoperative gradient (IR, 11.0 mmHg ± 3.4; ME, 10.58 mmHg ± 2.85; *P *= .6) showed no significant difference in the adjusted comparison.

Of the 104 patients included in the matched cohort, complete 3-year echocardiographic follow-up data were available for **52 (50%)**. The main reasons for loss of follow-up included patient relocation, missed follow-up appointments, and disruptions in routine echocardiography scheduling during the COVID-19 pandemic. A sensitivity analysis showed no significant baseline differences between patients with and without complete follow-up data.

Postoperative aortic insufficiency (AI) was trivial or absent in nearly all cases. One patient in the IR group developed mild AI at 1 year, with no worsening at 3 years. No patient required reintervention for AI.

### Mid-term clinical results

**Figure 1.**
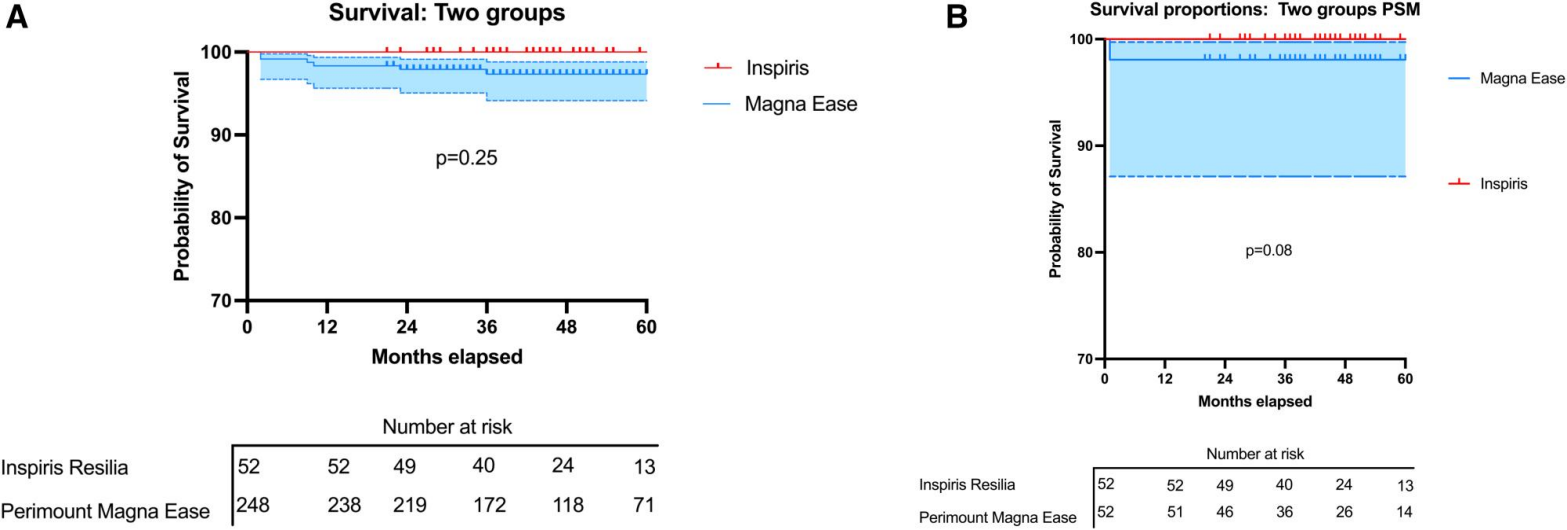
(A) Unadjusted survival probability estimate in the PERIMOUNT MAGNA EASE and the INSPIRIS RESILIA groups. (B) Propensity-matched survival probability estimates in the PERIMOUNT MAGNA EASE and the INSPIRIS RESILIA groups. Abbreviation: PSM, propensity score matching

Only one death in the adjusted population in the ME group. Survival in the IR group was 100% at discharge, 1 year, and overall. In the ME group, survival was 98.1% at discharge, at 1 year, and overall (**Figure 1**).

In the non-adjusted group, no deaths occurred in the IR group, whereas the ME group had an in-hospital mortality rate of 3.2%, a 1-year mortality rate of 4.4%, and 5.2% mortality over the study period (**Table 4**). No statistically significant survival advantage was observed for either bioprostheses.

**Table 4.** Survival Outcomes in the Unadjusted and in the Propensity-Matched Groups

	*Unadjusted*			*Propensity-matched*		
	INSPIRIS RESILIA (n = 52)	PERIMOUNT MAGNA EASE (n = 248)	*P-*value	INSPIRIS RESILIA (n = 52)	PERIMOUNT MAGNA EASE (n = 52)	*P-*value
In-hospital mortality	0 (0)	8 (3.2)	0.4	0 (0)	1 (1.9)	>.9
1-year mortality	0 (0)	11 (4.4)	0.2	0 (0)	1 (1.9)	>.9
Overall mortality	0 (0)	13 (5.2)	0.13	0 (0)	1 (1.9)	>.9

### Mid-term haemodynamic performance

At 1 year, the adjusted mean gradients were comparable (11.3 mmHg ± 3.56 in the IR group vs 11.2 mmHg ± 4.1 in the ME group; *P* = 0.9). Similarly, left ventricular ejection fraction was not significantly different between groups (IR: 60% ± 7 vs ME: 60% ± 6; *P* = 0.4). Regarding structural valve deterioration (SVD), one moderate case was observed in each group after adjustment (*P* > .9) (**Table 5**).

**Table 5.** One Year and 3 Years Echographic Outcomes in the Unadjusted and in the Propensity-Matched Groups

	*Unadjusted*			*Propensity-Matched*		
	INSPIRIS RESILIA (n = 52)	PERIMOUNT MAGNA EASE (n = 248)	*P-*value	INSPIRIS RESILIA (n = 52)	Perimount magna ease (n = 52)	*P-*value
**1-year outcomes**						
Mean gradient (mmHg)	11.3 ± 3.56	11.2 ± 4.0	.8	11.3 ± 3.56	11.2 ± 4.1	.9
LVEF (%)	60 ± 7	60 ± 8	.7	60 ± 7	60 ± 6	.4
PASP (mmHg)	27.3 ± 6.0	26.5 ± 5.3	.5	27.3 ± 6.0	26.1 ± 4.0	>.9
AI	1 (1.9)	0 (0)	.9	1 (1.9)	0 (0)	.9
SVD	1 (1.9)	1 (0.004)	.9	1 (1.9)	1 (1.9)	>.9
**3-year outcomes**						
Mean gradient (mmHg)	12.9 ± 3.7	12.8 ± 3.7	>.9	12.9 ± 3.7 (*n = *26)^a^	12.9 ± 3.6 (*n = *26)^a^	.7
LVEF (%)	59.6 ± 4.9	58.9 ± 6.1	.5	59.6 ± 4.9 (*n = *26)^a^	57.76 ± 5 (*n = *26)^a^	.16
PAPS (mmHg)	24.0 ± 3.9	26.1 ± 3.5	.10	24.0 ± 3.9 (*n = *26)^a^	26.9 ± 3.3 (*n = *26)^a^	.06
AI	0 (0)	0 (0)	–	0 (0) (*n = *26)^a^	0 (0) (*n = *26)^a^	–
SVD	1 (5.7)	2 (0.1)	0	1 (3.8) (*n = *26)^a^	1 (3.8) (*n = *26)^a^	>.9

Abbreviations: AI, aortic insufficiency; LVEF, left ventricular ejection fraction; PAPS, pulmonary artery systolic pressure; SVD, structural valve degeneration.

a Only patients with complete 3-year echocardiographic follow-up are included (*n* = 26 per group in the matched cohort).

**Figure 2.**
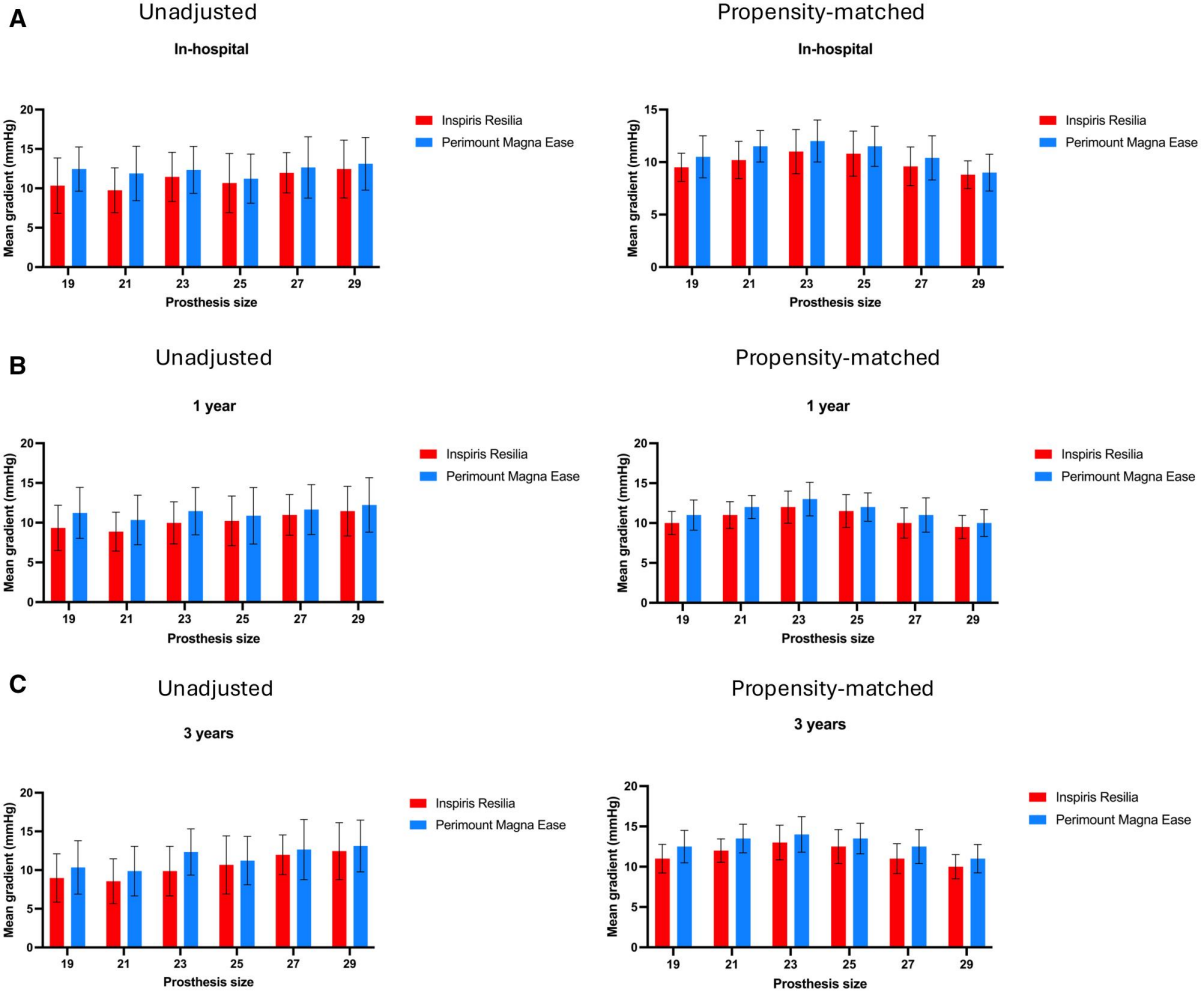
Mean Gradients in the INSPIRIS RESILIA and PERIMOUNT MAGNA EASE Groups in Both the Unadjusted and Propensity-Matched Populations, According to Prosthesis Size. (A) Before hospital discharge; (B) at 1 year postoperatively; and (C) at 3 years postoperatively

In the adjusted group, the highest mean gradient was noted in size 23 valves, followed by a decrease in gradient with increasing valve size (**Figure 2**, **Tables S1** and **S2**).

At 3 years, the adjusted mean gradient was slightly higher than at 1 year, recorded as 12.9 mmHg ± 3.7 in the IR group vs 12.9 mmHg ± 3.6 in the ME group (*P* = .7). No statistically significant differences were observed in other echocardiographic parameters. No new cases of SVD were identified compared to the 1-year results, and the 2 moderate SVD cases remained stable, without requiring surgical revision for AVR. At 3 years, in the adjusted population, the highest mean gradients were observed in size 23 valves, followed by a decrease in gradients with increasing valve size (**Figure 2**, **Tables S1** and **S2**). An interaction analysis was conducted to evaluate whether prosthesis size modified the relationship between prosthesis type and mean gradient. The interaction term was not statistically significant (*P* = .61), indicating that size alone was not a primary determinant of haemodynamic differences between these prostheses (**Figure S3**).

## DISCUSSION

This study demonstrates that the IR valve offers comparable mid-term clinical and haemodynamic outcomes to the ME valve in patients under 70 years of age undergoing surgical AVR. The main finding is the absence of significant differences in mortality, SVD, and transvalvular gradients at 1 and 3 years. The novelty of this study lies in its real-world design, the use of propensity score matching, and the focus on a younger patient population.

The rationale behind this comparison is that the ME valve has been the most widely implanted bovine pericardial valve globally for the past 15 years. Its haemodynamic stability has been extensively studied and proven by numerous studies.^7–9^ It also has demonstrated superiority over many other biological valves in terms of durability.^10^^,^^11^ The newer-generation **IR** is assembled from RESILIA tissue with anti-calcification properties. IR is also equipped with V-Fit stent technology, possibly beneficial for future transcatheter valve-in-valve implantation (TAVI ViV) procedures, which would allow for implantation of a larger size aortic valve than possible without this technology. However, studies on this topic are limited to case reports, which demonstrate the feasibility of this approach, with a CT scan confirming that the stent size increased from 22.2 mm to 24.2 mm after TAVI ViV.^12^^,^^13^

The most well-known studies supporting the IR valve are the COMMENCE studies. This study included patients (*n* = 689) with symptomatic aortic valve disease who underwent AVR with the **IR** bioprostheses. The study revealed low early mortality, low rates of paravalvular leak, and pacemaker implantation.^14^ The 5-year data from the study also confirmed the safety and good haemodynamic performance of the valve with RESILIA tissue in a predominantly low-risk population. This biological valve does not appear to undergo SVD, does not calcify unexpectedly, and is not prone to early thrombosis during the 5 years of observation.

A comparative study between IR and ME conducted by Francica et al, which compared 244 patients after propensity score matching, found similar haemodynamic performance results between the 2 groups at 1 and 3 years.^15^ Another large-scale comparative study conducted in Canada, involving 434 patients after propensity score matching, showed reassuring results in terms of safety and haemodynamic performance after 2 years of follow-up, with even better gradients in the INSPIRIS group.^16^ Additionally, in a non-comparative prospective French study involving 487 patients implanted with an IR bioprostheses reported a 1-year survival rate of 96.4% for all causes, with an endocarditis rate of 2.1%.^17^

Our study showed that IR aortic bioprostheses were safely implanted in 52 adult patients <70 years with severe aortic stenosis, insufficiency, or infectious endocarditis, yielding favourable clinical and haemodynamic outcomes up to 3 years. No mortality occurred in the IR group—hospital, 1-year, or overall—versus 1.9% in the ME group. One moderate SVD case occurred in each group at 1 year, remaining stable over 3 years without gradient worsening or surgical intervention. Two cases of infectious endocarditis arose in the IR group, none in the ME group (*P* = .5). Both cases occurred late, at 2 and 4 years postoperatively, and were classified as prosthetic valve endocarditis. The first case was caused by *Staphylococcus aureus* and the second by *Streptococcus sanguinis*. Neither was associated with intraoperative contamination or patient-related risk factors. These infections were most likely haematogenous in origin. Both patients underwent successful reoperation. These cases highlight that while the IR valve was not directly implicated.

Although more ME patients required reintervention, this difference did not reach statistical significance due to the small number of events. It may suggest a clinically meaningful signal that warrants further investigation in larger studies with longer follow-up. The relatively high reintervention rate in the ME group should be interpreted with caution, given the limited statistical power of our study.

The haemodynamic performance of the IR valve was similar to that of the ME valve at 1 year, and at 3 years. Analyses by valve size revealed no significant differences. The predominance of size 23 valves reflects the mean annular size and body surface area of our surgical population, with this size offering optimal fit in most cases. Valve sizing was based strictly on intraoperative sizer testing and not influenced by surgeon preference. Although the distribution of prosthesis sizes differed between the matched groups, an interaction analysis showed no significant effect of size on the relationship between prosthesis type and mean gradient, suggesting that prosthesis size did not confound the haemodynamic comparison.

The advantage of our study is that it reflects everyday clinical practice by including patients aged < 70 years with severe aortic stenosis, aortic insufficiency, or even endocarditis.

While mechanical valves remain the standard recommendation for younger patients due to their superior long-term survival, as shown in recent large cohort analyses,^18^ an increasing number of patients and heart teams are now opting for bioprostheses in this age group. This shift is largely driven by evolving patient preferences, the desire to avoid long-term anticoagulation, and advances in ViV technologies. According to the 2021 ESC/EACTS Guidelines,^6^ mechanical prostheses are still recommended for patients under 60 years of age. In our study, the choice of a bioprosthetic valve was based on multidisciplinary assessment that considered individual lifestyle, comorbidities, and anatomical factors. These findings contribute to current evidence on mid-term outcomes with tissue valves in younger adults and illustrate the increasing role of patient-centred decision-making in contemporary AVR strategies.

### Study limitations

The main limitation is the retrospective, non-randomized design and small sample size, which may reduce statistical power. Additionally, the relatively short follow-up period of 3 years limits the evaluation of long-term outcomes related to prosthesis-patient mismatch, which may become more apparent after 5 years, as shown in recent registry studies.^19^

Another major statistical limitation is missing data, particularly at the 3-year follow-up, where about 50% of echocardiographic data were unavailable, significantly reducing the robustness of long-term comparisons. Much of this is attributable to the COVID-19 pandemic, which disrupted routine follow-up and led to missing echocardiograms in eligible patients. Additionally, variations in echocardiographic protocols across centres may have caused inconsistencies in data collection. To assess the impact, we conducted a sensitivity analysis comparing baseline characteristics of patients with and without missing data. No significant differences were found, supporting the robustness of our conclusions.

Another limitation is the inclusion of patients undergoing concomitant procedures, which may introduce heterogeneity and potential bias. Although propensity score matching was applied, perfect balance was not achieved between groups.

Additionally, 3 redo cases were present in the IR group but none in the ME group, as redo surgery was not included in the propensity score model due to its low incidence. This residual imbalance may have introduced bias and should be considered when interpreting the findings.

Finally, the study lacks a comparative group receiving mechanical aortic valves, which limits broader conclusions regarding prosthesis selection in patients under 70 years of age.

## CONCLUSION

This study demonstrated that, in patients under 70 years of age, the INSPIRIS RESILIA aortic bioprosthesis offers comparable mid-term clinical outcomes to the PERIMOUNT MAGNA EASE valve, with no increase in morbidity or mortality observed up to 3 years post-implantation. Haemodynamic performance was similar between both groups, with stable mean transvalvular gradients at 1 and 3 years. The rate of SVD remained low and identical for both prostheses. Extended follow-up is necessary to confirm the long-term durability of the INSPIRIS RESILIA valve.

## Supplementary Material

ivaf169_Supplementary_Data

## Data Availability

Data are available after formal request to and acceptance by the Ethical Committee of Clinical Research of the French Society of Thoracic and Cardiovascular Surgery (comite-ethique@sfctcv.org).
