# Supplementary material for: Mid-Term Outcomes After Aortic Valve Replacement in Patients Under 70: A Comparative Study of INSPIRIS RESILIA Versus PERIMOUNT MAGNA EASE Bioprostheses
Source: Interdiscip Cardiovasc Thorac Surg. 2025 Jul 25;40(8):ivaf169. doi: 10.1093/icvts/ivaf169 (PMC12342774; doi:10.1093/icvts/ivaf169)

**Supplementary data**

**Table 1:** Mean gradients in the Inspiris Resilia and Perimount Magna Ease groups in the unadjusted population, according to prosthesis size.

| **Valve Size** | **In-hospital Unadjusted Inspiris Resilia** | **In-hospital unadjusted Magna Ease** | **1 Year Unadjusted Inspiris Resilia** | **1 Year unadjusted Magna Ease** | **3 years Unadjusted Inspiris Resilia** | **3 years Unadjusted Magna Ease** | **P-value** |
| --- | --- | --- | --- | --- | --- | --- | --- |
| 19 | 10.34 ± 1.49 | 12.45 ± 1.49 | 9.34 ± 1.45 | 11.23 ± 1.45 | 8.98 ± 1.57 | 10.34 ± 1.57 | 0.30 |
| 21 | 9.76 ± 1.23 | 11.89 ± 1.23 | 8.87 ± 1.10 | 10.34 ± 1.10 | 8.56 ± 1.19 | 9.87 ± 1.19 | 0.07 |
| 23 | 11.45 ± 0.82 | 12.34 ± 0.82 | 9.98 ± 0.76 | 11.45 ± 0.76 | 9.87 ± 0.96 | 12.34 ± 0.96 | 0.15 |
| 25 | 10.67 ± 1.16 | 11.23 ± 1.16 | 10.23 ± 1.15 | 10.87 ± 1.15 | 10.67 ± 1.16 | 11.23 ± 1.16 | 0.38 |
| 27 | 11.98 ± 1.44 | 12.65 ± 1.44 | 10.98 ± 1.23 | 11.65 ± 1.23 | 11.98 ± 1.44 | 12.65 ± 1.44 | 0.26 |
| 29 | 12.45 ± 1.94 | 13.12 ± 1.94 | 11.45 ± 1.83 | 12.23 ± 1.83 | 12.45 ± 1.94 | 13.12 ± 1.94 | 0,612984 |

**Table 2:** Mean gradients in the Inspiris Resilia and Perimount Magna Ease groups in the adjusted population, according to prosthesis size.

| **Valve Size** | **In-hospital Adjusted Inspiris Resilia** | **In-hospital Adjusted Magna Ease** | **1 Year Adjusted Inspiris Resilia** | **1 Year Adjusted Magna Ease** | **3 years Adjusted Inspiris Resilia** | **3 years Adjusted Magna Ease** | **P-value** |
| --- | --- | --- | --- | --- | --- | --- | --- |
| 19 | 9.50 ± 1.20 | 10.50 ± 1.20 | 10.00 ± 1.19 | 11.00 ± 1.19 | 11.00 ± 1.34 | 12.00 ± 1.34 | 0,306569 |
| 21 | 10.20 ± 0.78 | 11.50 ± 0.78 | 11.00 ± 0.74 | 12.00 ± 0.74 | 12.00 ± 0.69 | 13.00 ± 0.69 | 0,066026 |
| 23 | 11.00 ± 0.66 | 12.00 ± 0.66 | 12.00 ± 0.68 | 13.00 ± 0.68 | 13.00 ± 0.72 | 14.00 ± 0.72 | 0,155941 |
| 25 | 10.80 ± 0.59 | 11.50 ± 0.59 | 11.50 ± 0.56 | 12.00 ± 0.56 | 12.50 ± 0.58 | 13.50 ± 0.58 | 0,379891 |
| 27 | 9.60 ± 0.84 | 10.40 ± 0.84 | 10.00 ± 0.86 | 11.00 ± 0.86 | 11.00 ± 0.87 | 12.00 ± 0.87 | 0,260392 |
| 29 | 8.80 ± 0.99 | 9.00 ± 0.99 | 9.50 ± 0.95 | 10.00 ± 0.95 | 10.00 ± 1.00 | 11.00 ± 1.00 | 0,612984 |

**Figure 1:** Love plot


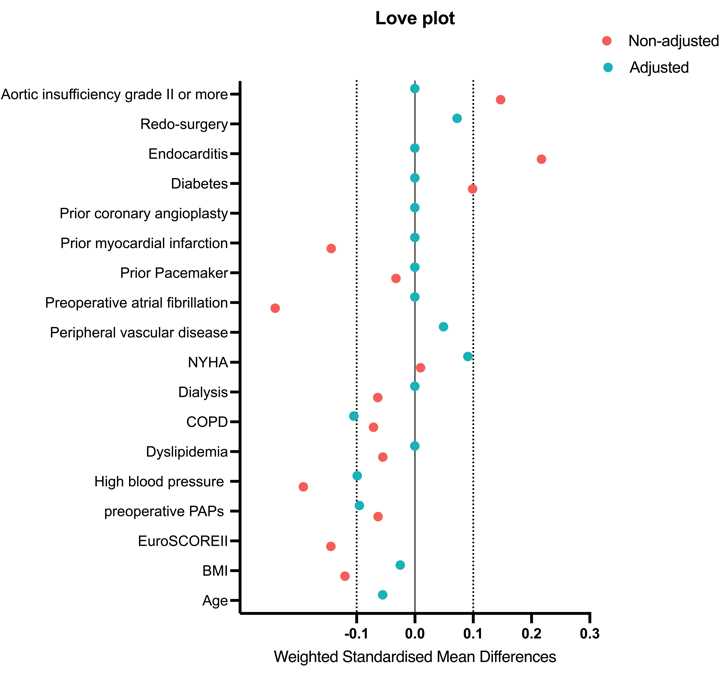

Supplement: ivaf169_Supplementary_Data [file ivaf169_supplementary_data.docx]
